# Begomovirus-Associated Satellite DNA Diversity Captured Through Vector-Enabled Metagenomic (VEM) Surveys Using Whiteflies (Aleyrodidae)

**DOI:** 10.3390/v8020036

**Published:** 2016-02-02

**Authors:** Karyna Rosario, Christian Marr, Arvind Varsani, Simona Kraberger, Daisy Stainton, Enrique Moriones, Jane E. Polston, Mya Breitbart

**Affiliations:** 1 College of Marine Science, University of South Florida, Saint Petersburg, FL 33701, USA; ckmarr@mail.usf.edu (C.M.); mya@usf.edu (M.B.); 2 School of Biological Sciences and Biomolecular Interaction Centre, University of Canterbury, Ilam, Christchurch 8041, New Zealand; arvind.varsani@canterbury.ac.nz (A.V.); simona.kraberger@pg.canterbury.ac.nz (S.K.); daisy.stainton@pg.canterbury.ac.nz (D.S.); 3 Department of Plant Pathology, University of Florida, Gainesville, FL 32611, USA; jep@ufl.edu; 4 Structural Biology Research Unit, Department of Clinical Laboratory Sciences, University of Cape Town, Rondebosch, Cape Town 7701, South Africa; 5 Instituto de Hortofruticultura Subtropical y Mediterránea ‘‘La Mayora’’ (IHSM-UMA-CSIC), Consejo Superior de Investigaciones Científicas, Estación Experimental ‘‘La Mayora’’, Algarrobo-Costa, Málaga 29750, Spain; moriones@eelm.csic.es

**Keywords:** begomovirus, begomovirus-associated satellites, alphasatellites, betasatellites, gammasatellites, Ipomoea satellites, metagenomics, whitefly, vector, ssDNA

## Abstract

Monopartite begomoviruses (*Geminiviridae*), which are whitefly-transmitted single-stranded DNA viruses known for causing devastating crop diseases, are often associated with satellite DNAs. Since begomovirus acquisition or exchange of satellite DNAs may lead to adaptation to new plant hosts and emergence of new disease complexes, it is important to investigate the diversity and distribution of these molecules. This study reports begomovirus-associated satellite DNAs identified during a vector-enabled metagenomic (VEM) survey of begomoviruses using whiteflies collected in various locations (California (USA), Guatemala, Israel, Puerto Rico, and Spain). Protein-encoding satellite DNAs, including alphasatellites and betasatellites, were identified in Israel, Puerto Rico, and Guatemala. Novel alphasatellites were detected in samples from Guatemala and Puerto Rico, resulting in the description of a phylogenetic clade (DNA-3-type alphasatellites) dominated by New World sequences. In addition, a diversity of small (~640–750 nucleotides) satellite DNAs similar to satellites associated with begomoviruses infecting *Ipomoea* spp. were detected in Puerto Rico and Spain. A third class of satellite molecules, named gammasatellites, is proposed to encompass the increasing number of reported small (<1 kilobase), non-coding begomovirus-associated satellite DNAs. This VEM-based survey indicates that, although recently recovered begomovirus genomes are variations of known genetic themes, satellite DNAs hold unexplored genetic diversity.

## 1. Introduction

Begomoviruses (family *Geminiviridae*) and their associated satellite DNAs, known as alphasatellites and betasatellites, form complexes that cause devastating diseases in agricultural systems [1,2,3,4]. These begomovirus-satellite complexes infect a wide range of dicotyledonous plants within at least 37 different genera in 17 families, spanning vegetable and fiber crops, ornamentals and uncultivated vegetation [2]. Diverse begomovirus-satellite complexes have been identified in all major dicotyledonous plant crops and evidence suggests that these complexes are rapidly expanding their host and biogeographical ranges, thus threatening agriculture in tropical and subtropical regions worldwide [1,3,4].

Begomoviruses contain circular single-stranded DNA (ssDNA) genomes that are either bipartite or monopartite; however, alphasatellites and betasatellites are predominantly associated with monopartite begomoviruses. Correspondingly, begomovirus-satellite complexes are thought to be mainly restricted to the Old World (OW) where monopartite begomoviruses outnumber those with bipartite genomes [5]. In particular, the highest diversity of both begomovirus species and satellite DNAs has been reported from China and the Indian subcontinent, which have been proposed as the centers of origin for these complexes [6]. Most of the disease complexes that have been described are composed of betasatellites and their helper begomoviruses. The majority of alphasatellites have been identified in plants infected with begomovirus-betasatellite complexes in the OW and, as a result, the known biogeographical distribution of alphasatellites is similar to that of betasatellites [7,8]. However, one disparity is the identification of alphasatellites associated with bipartite begomoviruses in the New World (NW) [9,10], whereas there are no official reports of natural begomovirus-betasatellite infections in the NW. The only begomovirus-betasatellite complex reported in the NW is a non-native complex infecting an ornamental plant that is propagated in a vegetative manner and, thus, does not represent a threat to agriculture [11,12].

Alphasatellites and betasatellites exhibit conserved features that share some broad characteristics. Both satellites consist of circular ssDNA molecules that are about half the size (~1350 nucleotides (nt)) of their helper begomovirus genomes [2,11]. These molecules both exhibit a putative origin of replication (*ori*) marked by a canonical nonanucleotide motif (NANTATTAC) at the apex of a predicted stem-loop structure [11,13]. However, betasatellites exhibit the same nonanucleotide motif observed in the overwhelming majority of begomoviruses (TAATATTAC), while alphasatellites exhibit the nonanucleotide motif seen in nanoviruses (family *Nanoviridae*) (TAGTATTAC) [7,11]. Both satellite DNA molecules are characterized by an adenine-rich region that may act, in part, as a “stuffer” to increase satellite size and allow efficient encapsidation and systemic movement by the helper begomovirus [2,11]. Alphasatellite and betasatellite DNAs each encode a single protein. Alphasatellite molecules encode a replication-associated protein (Rep) which allows this type of satellite to replicate autonomously. On the other hand, betasatellite molecules require a helper begomovirus for replication and encode a multifunctional protein, βC1, involved in disease pathogenicity [2,11,14,15]. In addition, betasatellite molecules share a common region approximately 120 nt long, known as the satellite common region (SCR), which spans the *ori* [12].

Alphasatellites and betasatellites are both dependent on helper begomoviruses for encapsidation, cell-to-cell and systemic movement within the host plant, and transmission by the whitefly vector, *Bemisia tabaci* [2,11]. However, the contributions of these two satellite types to the helper begomovirus and their role during begomovirus-satellite complex infections are distinct. Betasatellites are required for symptom induction and enhance helper begomovirus pathogenicity by increasing viral DNA levels in host plants and suppressing plant antiviral defense systems [2,11,16,17,18,19]. Alphasatellites, on the other hand, are thought to be non-essential for disease development or maintenance and, in some cases, have been shown to attenuate disease symptoms [11,20,21,22]. Some alphasatellites also encode a Rep with strong gene silencing suppression activity that may be more efficient at overcoming host defense mechanisms compared to betasatellites [22].

An interesting aspect of begomovirus-associated satellite DNAs is their degree of promiscuity. Both alphasatellites and betasatellites are capable of functionally interacting with different helper begomoviruses [2,11]. Although betasatellites require a helper begomovirus for replication, there are several examples of betasatellites that can be transreplicated by a diversity of non-cognate helper begomoviruses, including both monopartite and bipartite begomoviruses [2,4,23,24]. Furthermore, experimental evidence has shown that betasatellites can be transreplicated by beet curly top virus (BCTV), a curtovirus (*Geminiviridae*) not known to naturally associate with satellite molecules, with betasatellites complementing host defense suppression mechanisms of this non-cognate helper virus [20,25]. Despite the observed replicative promiscuity of betasatellites, phylogenetic analyses indicate that these molecules group according to the host from which they were originally isolated [12] and suggest that adaptation of betasatellites to their cognate helper begomoviruses for replication results from co-evolution [26]. In contrast, alphasatellites do not appear to have affinity to specific helper begomoviruses and, thus, may be relatively mobile [7,11]. For example, OW bipartite begomoviruses that are not naturally associated with alphasatellites can facilitate the systemic movement of these molecules in host plants [7,27,28]. Moreover, in laboratory experiments, the curtovirus BCTV successfully mediated the systemic movement of an ageratum yellow vein virus-associated alphasatellite and allowed the transmission of this satellite by the BCTV leafhopper vector, thus implying trans-encapsidation of the alphasatellite [27].

The formation of new disease complexes through the recruitment of satellite DNAs by unrelated begomoviruses during mixed infections presents a serious concern for agriculture [1,2,29]. Furthermore, a recent study described the detection of an unprecedented mastrevirus-alphasatellite-betasatellite complex in symptomatic wheat plants collected from fields in India, highlighting that satellite DNAs may also be naturally recruited by non-begomovirus species [30]. In addition, alphasatellite and betasatellite molecules may recombine, resulting in the emergence of new satellite DNAs [31]. The promiscuous nature of alphasatellites and betasatellites, coupled with the adaptive potential of begomoviruses due to their genome plasticity [32,33], may lead to the emergence of new damaging begomovirus-satellite complexes and pose a risk to agricultural regions not yet impacted by these disease complexes [2,4,11]. Therefore it is critical to gain a better understanding of the diversity and distribution of satellite DNAs.

This study surveyed begomoviruses and associated satellite DNAs present in whiteflies collected from multiple crops and native vegetation in several countries through vector-enabled metagenomics (VEM; where virus particles are purified and sequenced directly from insect vectors [34,35,36]). The VEM approach led to the detection of begomoviruses, including novel species, in all the locations [37]. Here we discuss the satellite DNAs identified through the VEM survey, which include several novel alphasatellites in the NW. In addition, this study expands a class of small satellite DNAs similar to previously sequenced satellites associated with begomoviruses infecting *Ipomoea* spp. Genomic features for these Ipomoea satellites are described and compared to the increasing number of reported small (<1 kilobase (kb)), non-coding begomovirus-associated satellite DNAs, named here gammasatellites.

## 2. Materials and Methods

### 2.1. Whitefly Collection, Sample Processing, Metagenomic Sequencing and Data Analysis

This study analyzes data from a recent VEM study investigating begomovirus diversity using whiteflies; thus, detailed methods have been previously published [37]. Briefly, adult whiteflies were collected from various crop fields and uncultivated native vegetation from geographically distant locations (Guatemala, Israel, Puerto Rico, Spain, and United States) using battery-operated vacuums and manual aspirators (Table 1). Whiteflies were collected from weeds (*Solanum nigrum*) as well as the following crop plants: bean (*Phaseolus vulgaris*), eggplant (*Solanum melongena*), pumpkin (*Cucurbita maxima*), squash (*Cucurbita pepo*), and tomato (*Solanum lycopersicum*). Whitefly specimens (100–350 whiteflies per field site) were homogenized in SM Buffer (50 mM Tris·HCl, 10 mM·MgSO_4_, 0.1 M NaCl, pH 7.5) and filtered through a 0.22 µm Sterivex filter (Millipore, Billerica, MA, USA) to partially purify virus particles before DNA extraction and sequencing. Sterivex filters were stored at −80 °C and used for a PCR assay to distinguish between different whitefly species and *B. tabaci* phylogenetic groups based on the mitochondrial cytochrome c oxidase I gene [38]. DNA was extracted from 200 µL of filtrate using the QIAmp MinElute Virus Spin Kit (Qiagen, Valencia, CA, USA) following manufacturer’s instructions and used as template for rolling circle amplification (RCA) using the illustra TempliPhi DNA Amplification Kit (GE Healthcare, Little Chalfont, Buckinghamshire, UK) to enrich for small circular genomes and DNA molecules [39]. Six replicate RCA reactions were performed for each sample. RCA replicates for each sample were pooled before sequencing through multiplexing at a commercial facility using the 454 GS FLX System (Roche, Indianapolis, IN, USA).

Metagenomic reads (average read length 263 nt) from each sample were dereplicated using default settings in the CD-Hit web server [40]. Only reads longer than 100 nt were used for assembly (minimum identity of 98% over 35 nt) using Geneious version R7 (Biomatters, Newark, NJ, USA). Both contigs and unassembled reads were compared against the GenBank non-redundant database using BLASTn and BLASTx (*e*-value < 0.001) [41]. Sequences with matches to begomovirus-associated sequences, including satellite DNAs, were identified and sorted using the Metagenome Analyzer (MEGAN4) software [42]. Analyzed contigs and unassembled reads generated from the different libraries are publicly available on the METAVIR web server [43] under project names “Whiteflies” and “Whiteflies_Unassembled”.

**Table 1:** Whitefly samples used for vector-enabled metagenomic (VEM) survey of begomovirus-associated satellite DNAs [37].

Crop/Plant (Dataset) ^1^	Location	Collection Date	Whiteflies ^2^
Squash	Imperial Valley, California, USA	June 2012	*B. tabaci* (MEAM1)
Tomato	Salamá, Baja Verapaz, Guatemala	January 2012	*B. tabaci* (NW), *T. vaporariorum*
Tomato (Guat_T2)	El Progreso, Jutiapa, Guatemala	January 2012	*B. tabaci* (NW), *T. vaporariorum*
Squash	Teculután, Zacapa, Guatemala	January 2012	*B. tabaci* (MEAM1)
Bean	Torrox, Málaga, Spain	September 2011	*B. tabaci* (MED)
Squash (Spain_S)	Torrox, Málaga, Spain	September 2011	*B. tabaci* (MED)
Tomato (Spain_T)	Torrox, Málaga, Spain	September 2011	*B. tabaci* (MED)
European black nightshade	Algarrobo-Costa, Málaga, Spain	September 2011	*B. tabaci* (MED)
European black nightshade	Algarrobo-Costa, Málaga, Spain	September 2011	*B. tabaci* (MED)
Tomato	Bet Dagan, Israel	March 2011	*B. tabaci* (MEAM1)
Squash (Israel_S)	Bet Dagan, Israel	March 2011	*B. tabaci* (MEAM1)
Tomato (PR_T1)	Santa Isabel, Puerto Rico	November 2010	*B. tabaci* (MEAM1)
Tomato (PR_T2)	Santa Isabel, Puerto Rico	November 2010	*B. tabaci* (MEAM1)
Pumpkin (PR_P)	Santa Isabel, Puerto Rico	November 2010	*B. tabaci* (MEAM1)
Eggplant (PR_E)	Santa Isabel, Puerto Rico	November 2010	*B. tabaci* (MEAM1)

^1^ Indicates the crop or weed species from which whitefly specimens were collected. Dataset IDs for metagenomic libraries containing satellite DNA sequences are specified in parentheses. Highlighted samples indicate locations where begomovirus-associated satellite DNAs were identified. Colors distinguish if novel (green) or known (grey) satellite DNAs were detected; ^2^ Whitefly species (*Bemisia tabaci* or *Trialeurodes vaporariorum*) used for VEM were classified based on phylogenetic groups including the Middle East-Asia Minor 1 (MEAM1), Mediterranean (MED), and New World (NW) clades.

### 2.2. Satellite DNA Molecule Completion

Contigs and/or unassembled reads potentially representing new begomovirus-associated satellite DNAs, including alphasatellites (<83% sequence identity to known species; [44]), betasatellites (<78% sequence identity to known species; [45]), and small (<1 kb) satellite DNAs, were used to design back-to-back (abutting) primers for inverse PCR assays to recover full-length DNAs. Inverse PCRs were performed using the HerculaseII Fusion DNA Polymerase (Agilent Technologies, Santa Clara, CA, USA) and products were cloned using the CloneJET PCR Cloning Kit (Thermo Scientific, Waltham, MA, USA). All clones were commercially Sanger sequenced with a minimum of 2× coverage using vector primers and primer walking. Satellite DNAs were assembled using the Sequencher software (Gene Codes Corporation, Ann Arbor, MI, USA) and final sequences were inspected using SeqBuilder from the Lasergene software package (DNASTAR, Madison, WI, USA). Open reading frames (ORFs) encoding putative proteins > 70 amino acids were compared against the GenBank non-redundant database for annotation purposes.

### 2.3. Satellite DNA Sequence Analysis

Satellite DNA sequences were compared against reported sequences in GenBank using pairwise comparisons, multiple sequence alignments, and phylogenetic trees. All pairwise comparisons were performed using the MUSCLE algorithm [46] implemented in the Species Demarcation Tool (SDT) version 1.2 [47]. Multiple sequence alignments were performed using the MUSCLE algorithm implemented in the MEGA5 software [48] and edited manually. A maximum likelihood (ML) phylogenetic tree was constructed to evaluate the relationship among divergent alphasatellite sequences reported here and those found in the database (*n* = 84). For this purpose, predicted Rep amino acid sequences were aligned and a ML phylogenetic tree was constructed with the best-fit model (LG + I + G + F) according to ProtTest [49] using the PhyML server [50] with the approximate likelihood ratio test (aLRT) to assess branch support [51]. Branches with <70% support were collapsed using TreeCollapserCL4 [52].

## 3. Results

### 3.1. Overview

VEM was used to investigate the diversity of begomovirus-associated satellite DNAs circulating in various crop fields and uncultivated vegetation from four countries (Guatemala, Israel, Spain, continental United States (California)) and an island in the Caribbean (Puerto Rico) (Table 1). Pyrosequencing of RCA-amplified viral nucleic acids from whiteflies and BLAST analysis of assembled contigs and unassembled sequence reads allowed for the detection of satellite DNAs in eight out of fifteen metagenomic datasets, with satellites present in all the locations except California (USA) (Table 1). Whiteflies sampled from most crop plants, except for beans, revealed the presence of satellite DNAs in at least one location. Satellite DNAs were not detected in whiteflies collected from weeds; however, these samples only originated from a single species (*Solanum nigrum*) in a single location. Seventy two circular satellite-like DNAs, ten of which were verified through inverse PCR, were assembled and completed (Table 2). Sixty two of these completed satellite DNAs represent a novel class of small satellites similar to satellites associated with begomoviruses infecting *Ipomoea* spp., named Ipomoea satellites (Unpublished, GenBank accession numbers FJ914390–FJ914405). We propose naming these small (<1 kb), non-protein-coding satellite DNAs as “gammasatellites” to distinguish them from the previously described alphasatellites and betasatellites.

### 3.2. Detection of Protein-Encoding Satellite DNAs and Identification of a New Alphasatellite Clade

Protein-encoding satellite DNA molecules, including alphasatellites and betasatellites, were identified in whiteflies collected in Israel, Puerto Rico, and Guatemala (Table 2). Whiteflies collected from squash in Israel contained an alphasatellite molecule that was most similar to an alphasatellite associated with Cotton leaf curl Gezira (CLCuGe) virus (96% identity) initially identified in Burkina Faso, West Africa [53]. The VEM CLCuGe alphasatellite (1359 nt in size) exhibits an organization similar to that of the African alphasatellite. In addition, multiple contigs from the Israel squash dataset had similarities to betasatellite molecules associated with monopartite begomoviruses infecting okra, named CLCuGe betasatellites or CLCuGeB [45]. Three contigs representing CLCuGeB circular DNAs were assembled, one of which was verified by PCR. These VEM CLCuGeB sequences shared 96%–98% pairwise identity with CLCuGeB molecules identified in Egypt. Although CLCuGe virus was identified in the same metagenomic dataset from Israel [37], it is difficult to establish a direct association between these satellite molecules and their helper viruses in individual host plants. Most of the sequences similar to the VEM CLCuGe alphasatellite assembled into a single contig. In contrast, there were multiple contigs and unassembled sequence reads similar to CLCuGeB, indicating a higher genetic variability for betasatellites than alphasatellites at this location. This is consistent with studies investigating begomovirus-satellite complexes in crops which describe a higher variability of betasatellites than alphasatellites (e.g., [5,8,53]).

**Table 2:** Satellite DNA molecules detected in whiteflies.

Dataset	Genome ^a^ (Accession Number)	Best Match ^b^ (% Identity)/Accession Number	Satellite Size (nt)
Guat_T2	VEM alphasatellite 1a (KT099170)	MeCMV-associated alphasatellite, Venezuela (76%)/KF670648	1370
	VEM alphasatellite 1b (KT099171)	MeCMV-associated alphasatellite, Venezuela (74%)/KF670648	1329
	VEM alphasatellite 2 (KT099172)	Ageratum enation virus associated DNA 1, India (71%)/FN543100	1359
Israel_S	VEM Cotton leaf curl Gezira betasatellite * (KT099176)	Cotton leaf curl Gezira betasatellite, Egypt(95%)/AF397215	1324
	VEM Cotton leaf curl Gezira betasatellite * (KT099177)	Cotton leaf curl Gezira betasatellite, Egypt (99%)/AF397217	1348
	VEM Cotton leaf curl Gezira betasatellite (KU095847)	Cotton leaf curl Gezira betasatellite, Egypt(98%)/AF397215	1310
	VEM Cotton leaf curl Gezira virus alphasatellite * (KT099178)	Cotton leaf curl Gezira virus DNA 1, Burkina Faso (96%)/FN554581	1359
PR_T1	VEM Ipomoea begomovirus satellite 1 (KT099179)	Ipomoea begomovirus satellite DNA beta isolate, Spain (70%)/FJ914403	731
	VEM Ipomoea begomovirus satellite 2 (KT099180)	Ipomoea begomovirus satellite DNA beta isolate, Spain (70%)/FJ914403	713
	VEM Ipomoea begomovirus satellite 3 (KT099181)	SPLCLaV replication-associated protein, Spain (95%) **/EU839579	743
	VEM alphasatellite 3a (KT099173)	Cuban alphasatellite 1, Cuba (86%)/HE806451	1300
PR_P	VEM alphasatellite 3b (KT099174)	Cuban alphasatellite 1, Cuba (85%)/HE806451	1307
	VEM alphasatellite 3c (KT099175)	Cuban alphasatellite 1, Cuba (86%)/HE806451	1303
PR_T1, T2, P, and E	VEM Ipomoea begomovirus satellite (Contigs, n = 49) *	Ipomoea begomovirus satellite DNA beta isolate, Spain (< 80%)/Various	
Spain T and S	VEM Ipomoea begomovirus satellite (Contigs, n = 10) *	Ipomoea begomovirus satellite DNA beta isolate, Spain (> 90%)/Various	

^a^ Genomes that were not PCR-verified are highlighted with an asterisk (*). Genomes highlighted in grey summarize multiple Ipomoea satellites identified in datasets from Puerto Rico and Spain (Contig sequences representing complete Ipomoea satellites are provided in Supplemental File S1). ^b^ Genome-wide pairwise identities for best match in GenBank. Abbreviations refer to melon chlorotic mosaic virus (MeCMV) and sweet potato leaf curl Lanzarote virus (SPLCLaV). ** The best GenBank match to VEM Ipomoea begomovirus satellite 3 was a portion of the Rep-encoding region of the SPCLaV genome and the reported identity is only within this small region (8% coverage).

**Figure 1:**
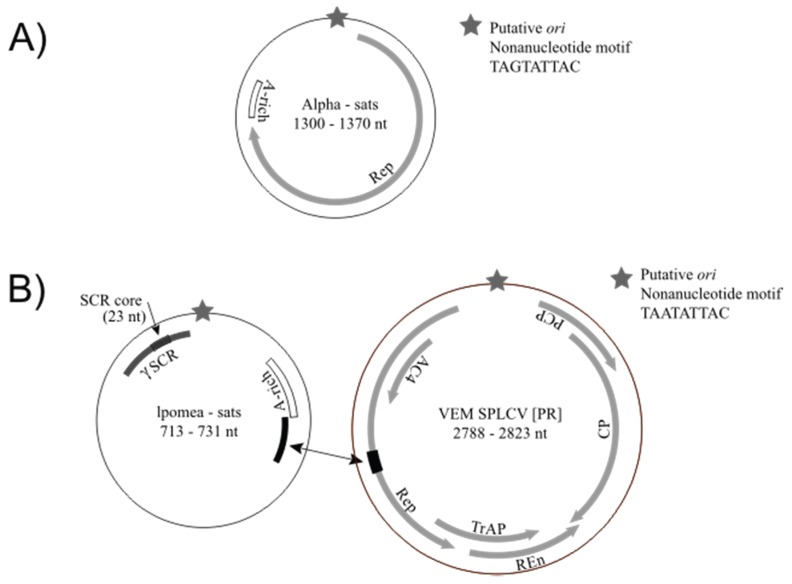
Schematics depicting general genome organization and features observed in novel alphasatellites (**A**) and Ipomoea satellites (**B**) detected during this study. All genomes are characterized by a putative origin of replication (*ori*) marked by a stem-loop structure containing a conserved nonanucleotide motif. Alphasatellites encode a replication-associated protein (Rep) and exhibit an adenine-rich (A-rich) region. Ipomoea satellites do not exhibit any coding regions; however, these gammasatellites contain an A-rich region as well as a conserved region ~100 nt long shared among various gammasatellites (γSCR). The γSCR contains a 23 nt stretch where all gammasatellites reported to date share high similarities with betasatellites, named here the satellite common region (SCR) core. Some Ipomoea satellites from Puerto Rico share high identities with a 66 nt stretch (highlighted in black) found within the *rep* gene of sweet potato leaf curl virus (SPLCV) genomes detected in the same region.

The VEM approach also enabled the detection of novel alphasatellite molecules in Guatemala and Puerto Rico, which exhibit typical features of alphasatellite DNAs (Table 2, Figure 1A). Since alphasatellites have rarely been found in the NW [9,10] and detected sequences are divergent from known alphasatellites, all NW alphasatellites were verified by PCR. The alphasatellites detected in Guatemala represent the first alphasatellite DNAs reported from Central America. Three sequences representing two species of alphasatellite molecules, named VEM alphasatellites 1 and 2, were sequenced from one of the Guatemala tomato field sites. The VEM alphasatellite 1 sequence shares approximately 64% identity with that of VEM alphasatellite 2 and both satellite molecules are most similar (~70%–75% identity) to alphasatellite molecules from India. In addition, three alphasatellite molecules representing a single species, named VEM alphasatellite 3, were sequenced from samples collected from tomato and pumpkin fields in Puerto Rico. The VEM alphasatellite 3 molecules range in size from 1300 to 1307 nt and share 93%–99% identity to each other. These molecules are most similar (~85% identity) to an alphasatellite molecule, Cuban alphasatellite 1, recently identified in weeds from Cuba [54]. In general, VEM alphasatellite 3 has a similar organization to Cuban alphasatellite 1, with one major ORF encoding a Rep and an adenine-rich region. However, Cuban alphasatellite 1 has two additional predicted ORFs with coding capacity > 100 amino acids, one overlapping the Rep ORF and another one identified in the complementary strand. All of the VEM alphasatellite 3 molecules also have the ORF overlapping the Rep; however, an ORF on the complementary strand similar in sequence to the one in Cuban alphasatellite 1 was not identified in any of the VEM alphasatellite 3 DNAs.

**Figure 2:**
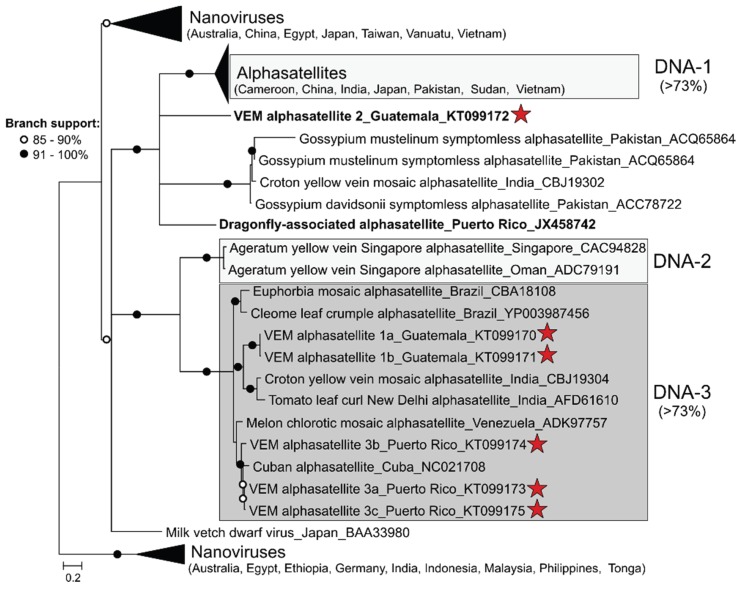
Midpoint-rooted maximum likelihood phylogenetic tree of predicted alphasatellite and nanovirus replication-associated protein sequences. Alphasatellites detected during this study are highlighted with a red star. Three groups of alphasatellites based on amino acid pairwise-identities are highlighted in shades of grey, including DNA-1-type alphasatellites from the Old World, DNA-2-type, and DNA-3-type alphasatellites mainly identified in the New World. Branches exhibiting less than 70% branch support were collapsed. Branches with approximate likelihood ratio test (aLRT) support > 91% are indicated with black circles, whereas branches exhibiting 85%–90% support are marked with a white circle. A list of sequences used for phylogenetic analysis and Rep pairwise identities are provided in Supplemental File S2.

Phylogenetic analysis of predicted alphasatellite Rep amino acid sequences indicates that the NW alphasatellites reported here and those reported from South America [9,10] and the Caribbean [54,55] do not cluster together (Figure 2). However, the Rep sequences of most NW alphasatellites share >73% amino acid pairwise identity and form a clade. This “NW alphasatellite-dominated” clade is most closely related (51%–55% amino acid identity) to ageratum yellow vein Singapore alphasatellite (AYVVSGA) molecules from Singapore and Oman, which were originally described as DNA-2 [21,27]. These “DNA-2-type” alphasatellites are phylogenetically distinct from other OW alphasatellites formerly known as DNA-1 [27]. Since the “NW alphasatellite-dominated” clade is also distinct from DNA-2-type alphasatellites, we have designated members of this clade as DNA-3-type alphasatellites. In addition to alphasatellites identified in the NW, the DNA-3-type alphasatellite clade contains two unique Rep sequences from alphasatellites reported from India, including croton yellow vein mosaic alphasatellite which is closely related to alphasatellites reported from Brazil and Venezuela [56]. There are two NW alphasatellite sequences that are distantly related to those of the DNA-3-type alphasatellite clade (shown in bold font in Figure 2), instead being more closely related to DNA-1-type alphasatellites from the OW. One of these sequences was detected in whiteflies collected from tomato plants in Guatemala (VEM alphasatellite 2) and the other was reported from dragonflies collected in an agricultural field in Puerto Rico (Dragonfly-associated alphasatellite; [55]). Therefore, these data demonstrate that more than one “type” of alphasatellite molecule is present in the NW.

DNA-2- and DNA-3-type alphasatellites share characteristics that are absent in other alphasatellites. One of the first alphasatellites detected in the NW, DNA-3-type melon chlorotic mosaic virus alphasatellite, contains a putative ORF encoding >100 amino acids that overlaps the *rep* [9]. Both DNA-2-type and DNA-3-type alphasatellites, except for euphorbia mosaic alphasatellite (Brazil), contain this putative ORFwhereas this ORF is not present in alphasatellites outside this group. It is currently unknown if this ORF is expressed and what role, if any, its product plays in infectivity. Finally, DNA-2- and DNA-3-type alphasatellites have a 13 amino acid insertion in the Rep C-terminus that is absent in all other alphasatellite Reps. This unique 13 amino acid stretch was originally noted in alphasatellites from Brazil and Singapore [10]; however, our analysis suggests that this insertion is a common feature of DNA-2- and DNA-3-type alphasatellite Reps.

### 3.3. Detection of Non-Coding Small Satellite DNAs (Gammasatellites)

**Figure 3:**
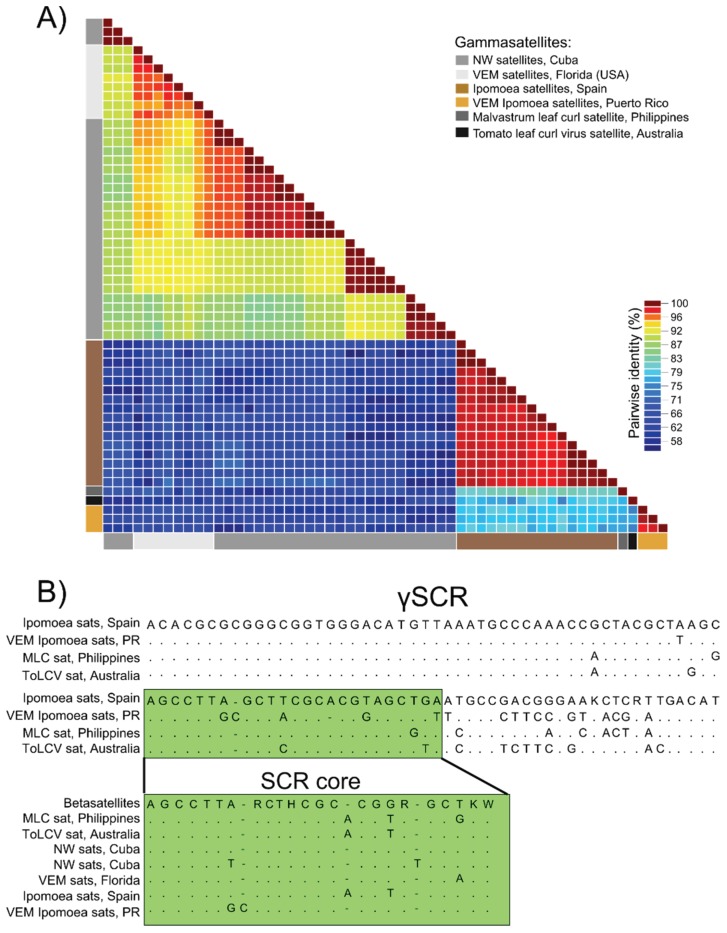
(**A**) Two-dimensional color-coded matrix depicting genome-wide pairwise identities observed among gammasatellites and (**B**) consensus sequences observed within a conserved satellite common region shared among various gammasatellites (γSCR). Mixed bases in the consensus sequences include K (G/T), R (A/G), W (A/T), and H (A/C/T). The γSCR was observed in Ipomoea satellites (Ipomoea sats) as well as tomato leaf curl virus sat (ToLCV sat) and malvastrum leaf curl satellite (MLC sat). A conserved 23 nt stretch (positions 663–686; labelled SCR core) found within the γSCR (positions 613–711) shares high pairwise identities with betasatellites and all gammasatellites reported to date, including novel New World satellites (NW sats) identified in Cuba and satellites identified in whiteflies from Florida, USA (VEM sats). Nucleotide positions are based on VEM Ipomoea begomovirus satellite 1. A list of sequences used for calculating identity scores among gammasatellites as well as betasatellites used for investigating the SCR core is provided in Supplemental File S3.

A novel class of small (~640–750 nt in size) satellite DNAs associated with begomoviruses infecting *Ipomoea* spp., named Ipomoea satellites, were detected in samples from Spain and Puerto Rico (Table 2). Non-coding begomovirus-associated satellite DNAs smaller than 1 kb have been increasingly identified in the past few years [35,57]. Here we propose that these small non-coding satellite DNAs represent a third class of satellite DNAs, named “gammasatellites”, that are distinct from alphasatellites and betasatellites, which are both protein-coding satellite DNAs >1 kb. Ten satellite sequences assembled from the tomato and squash datasets from Spain had high sequence pairwise identities (>94%) with Ipomoea satellite DNA sequences previously reported from Spain (accession numbers FJ914390–FJ914405). Additionally, fifty-two satellite DNA sequences assembled from the four Puerto Rico datasets shared more than 91% identity to each other but only 60% to 70% similarity to Ipomoea satellite DNA sequences from Spain (Figure 3A). Since little is known about Ipomoea satellites, three molecules recovered from Puerto Rico were verified through PCR and compared to assembled, full-length Ipomoea satellite sequences to identify conserved features.

Ipomoea satellites reported to date contain several conserved features, including the key characteristics of gammasatellites, which are a size <1 kb and the lack of recognizable ORFs (Figure 1B). Ipomoea satellites, like other gammasatellites reported to date, exhibit a putative *ori* marked by the canonical nonanucleotide motif TAATATTAC at the apex of a putative stem loop structure similar to that found in begomoviruses and betasatellites. Similar to other begomovirus-associated satellites [2,11], Ipomoea satellites contain an adenine-rich region. Ipomoea satellites from Spain have ~50% adenines over a 200 nt stretch whereas this region in satellites from Puerto Rico encompasses 60–100 nt. In addition, all Ipomoea satellites contain a region where they share more than 80% identity over 100 nt (Figure 1B and Figure 3B). This conserved region is also found in other gammasatellites reported from tomato crops in Australia (tomato leaf curl virus satellite, ToLCV-sat; [58]) and weeds in the Philippines (malvastrum leaf curl virus—associated satellite, MaLCV-sat; accession number KF433066); however, this region is absent in gammasatellites reported from Florida through VEM (VEM-sats) [35] and weeds from Cuba [57]. Pairwise analysis of full-length gammasatellite molecules indicate that molecules containing the conserved 100 nt stretch share higher identities with each other than to those that lack this conserved stretch (Figure 3A).

Since more than one type of satellite DNA seems to share a satellite common region (SCR), which has been traditionally associated with betasatellites [11], here we distinguish between the SCRs of gammasatellites and betasatellites. We refer to the common region spanning ~120 nt that is shared among the majority of betasatellites [12] as the βSCR, whereas the 100 nt long region shared among several gammasatellites is referred to as the γSCR. Embedded within the γSCR there is a 23 nt stretch that is highly conserved (87%–100% identity) among all gammasatellites reported to date. This 23 nt long region was originally identified in gammasatellites from Cuba based on its high identity (95.7%) with a portion of the βSCR [57] (Figure 3B).

Sequences representing sweet potato-infecting begomoviruses (sweepoviruses) were also identified in all the datasets containing Ipomoea satellite sequences [37]. Further work would be needed to confirm which helper begomoviruses are associated with the Ipomoea satellites identified in Puerto Rico and Spain. However, it is notable that approximately half of the Ipomoea satellite molecules identified from Puerto Rico share 89% pairwise identity with a small region (66 nts) of the *rep* gene of the three sweepovirus genomes identified in samples from the island (Figure 1B). This shared region potentially links some of the Ipomoea satellites with sweet potato leaf curl virus (SPLCV). Since this region is not a conserved feature among the identified satellites from Puerto Rico, it may not be a crucial component for the biology of these molecules. Moreover, a similar region was not identified in known SPLCV genomes and reported Ipomoea satellites from Spain.

## 4. Discussion

Here we implemented the VEM approach to survey begomovirus-associated satellite DNAs circulating in crop fields and amongst weeds located in different countries by sampling their whitefly vector. The VEM survey resulted in the detection of satellite DNAs in most of the locations and further expanded the known diversity of these molecules. While satellite DNA molecules have traditionally been associated with OW monopartite begomoviruses, recent studies have identified alphasatellite and novel gammasatellite molecules associated with bipartite begomoviruses in the NW, indicating that satellite DNAs are more widespread than previously thought [9,10,54,55]. All three types of satellite DNAs, namely alphasatellites, betasatellites, and gammasatellites, were identified amongst the OW samples investigated in this study. Both alphasatellites and betasatellites potentially associated with CLCuGe virus were identified in Israel. Ipomoea satellites found in Spain were highly similar to sequences deposited into GenBank from the same regions (accession numbers FJ914390−FJ914405). Although no novel satellite DNAs were detected in samples from the OW, the VEM approach revealed the presence of novel alphasatellites in Puerto Rico and Guatemala as well as unique Ipomoea satellites in all the samples collected in Puerto Rico.

Including the sequences described in this study, there are currently 11 known NW alphasatellites, suggesting that alphasatellite molecules are widespread in the NW. These alphasatellites represent six species according to the suggested 83% pairwise identity demarcation criteria for alphasatellites [44]. These NW alphasatellites have been identified from symptomatic plants in South America (Brazil and Venezuela) [9,10] and Cuba [54], as well as in insects, including alphasatellites accumulated in whiteflies (present study) and top insect predators (dragonflies) collected in Guatemala and Puerto Rico [55]. Based on the few available sequences, individual NW alphasatellites may have a fairly limited geographic distribution. VEM alphasatellites 1 and 2, representing two new species, were only detected in whiteflies collected from a single tomato field in Guatemala. Similarly, none of the alphasatellites reported from South America have been identified in more than one country. However, VEM alphasatellite 3 detected in Puerto Rico may represent the same species as Cuban alphasatellite 1. The Cuban alphasatellite 1 was only identified in a single plant and it was speculated that there was a specific association between tomato yellow leaf distortion virus (ToYLDV) and the Cuban alphasatellite in *Sida* plants [54]. However, VEM alphasatellite 3 sequences were detected in whiteflies feeding on tomato and pumpkin in farms separated by ~2.3 kilometers. Moreover, only contig sequences with low identities (~80% identity) to ToYLDV DNA-B were identified in metagenomic libraries from Puerto Rico [37]. Therefore, VEM alphasatellite 3 may associate with a different strain of ToYLDV or a different begomovirus species. Additional studies focused on identifying alphasatellites in the NW are needed to determine the biogeography of these molecules and identify their helper viruses.

Phylogenetic analysis of Rep amino acid sequences revealed that NW alphasatellites reported to date are not monophyletic (Figure 2). Although NW alphasatellites do not form a monophyletic group, most sequences reported from the NW form a well-supported clade, named here DNA-3-type alphasatellite clade, and share >73% identity. The alphasatellites encompassing the DNA-3-type clade are dominated by sequences from the NW and are most closely related to sequences representing AYVVSGA, with which they share genomic features not found in other alphasatellites. AYVVSGA has been identified as an “unusual” alphasatellite due to the shorter size (295 amino acids) and low sequence identity (<53% sequence identity) of its Rep compared to the Reps encoded by other alphasatellite molecules [7,27]. Therefore AYVVSGA was named DNA-2 [27] to distinguish this divergent satellite molecule from other OW alphasatellites (formerly known as DNA-1, [59]) which share more than 73% identity among each other (Figure 2). Despite the similarities observed between the DNA-2- and DNA-3-type alphasatellite sequences, DNA-2-type alphasatellites are unique in comparison to DNA-3-type alphasatellites identified in the NW. Reps encoded by DNA-3-type alphasatellites are ~315 amino acids long and share 51–55% amino acid identity with AYVVSGA, which make these alphasatellites as distinct from DNA-2-type alphasatellites as these molecules are from OW DNA-1-type alphasatellites. Furthermore, in contrast to both DNA-1- and DNA-2-type alphasatellites, DNA-3-type alphasatellites for which the helper virus is known are only associated with bipartite begomoviruses in infected plants [9,10,54]. Interestingly, the DNA-2-type alphasatellite is the only one that has been shown to associate with monopartite and bipartite begomoviruses from the OW and NW, respectively, in an experimental setting [27]. Therefore the DNA-2-type AYVVSGA may represent an intermediate species between OW DNA-1-type and NW DNA-3-type alphasatellites.

The identification of the DNA-3-type alphasatellite clade with unique characteristics that differ from OW alphasatellites raises the possibility that there are alphasatellites that are indigenous to the NW and, thus, have a different evolutionary history compared to their OW counterparts. It should be noted that the DNA-3-type alphasatellite clade includes two sequences from India. If this clade indeed represents a NW lineage of alphasatellites, this would suggest that the DNA-3-type alphasatellites from India represent NW satellite DNAs imported to Asia. Alternatively, DNA-3-type alphasatellites may have spread to the NW from the OW. There are two NW alphasatellites, VEM alphasatellite 2 from Guatemala (present study) and Dragonfly-associated alphasatellite from Puerto Rico [55], that are distantly related to the DNA-3-type alphasatellite clade. Therefore it is likely that there have been at least two independent events that led to the emergence of alphasatellites in the NW. These two divergent alphasatellites are more closely related to alphasatellites identified in Africa, Asia, and the Middle East, including DNA-1-type alphasatellites, than they are to the DNA-3-type alphasatellite clade (Figure 2). Further sampling is needed to help resolve the DNA-3-type alphasatellite clade and evaluate if divergent NW alphasatellites that are more similar to OW alphasatellites have been introduced from the OW or represent different NW alphasatellite lineages. Since alphasatellites from both the DNA-3-type alphasatellite clade and those similar to OW alphasatellites have been identified in the same regions (*i.e.*, Guatemala and Puerto Rico), geographic location is not the only factor influencing the distribution of alphasatellites in the NW.

A further distinction between NW and OW alphasatellites is that OW alphasatellites are mainly associated with begomovirus-betasatellite complexes [5,8,11,12], whereas NW alphasatellites do not seem to be associated with betasatellites. There are no reports of betasatellites in the NW, even though studies have specifically searched for betasatellite DNAs while investigating NW alphasatellites in plant specimens [9]. Furthermore, deep sequencing of RCA products recovered from plants [54] and whiteflies (present study) have not revealed the presence of betasatellites. It is possible that NW alphasatellites associate with divergent satellite DNAs that may not be recognized through standard BLAST searches. Alternatively, NW alphasatellites may not be associated with other satellite DNAs, solely relying on their helper begomoviruses [10].

Finally, this study significantly increased our knowledge of gammasatellites, which are non-coding begomovirus-associated satellite DNAs smaller than 1 kb. Although a discernable role in disease development has not yet been observed for gammasatellites, these satellite DNAs seem to be widespread since they have been previously reported from Australia [58], Philippines (Accession Number NC021929), Spain (accession numbers FJ914390–FJ914405), Cuba [57], and Florida [35]. A subset of gammasatellites, named Ipomoea satellites, was detected in samples from Spain and Puerto Rico. Ipomoea satellites from Puerto Rico exhibited the highest genetic diversity and were distinct from satellites detected in Spain. Analyses of Ipomoea satellites detected here and those previously reported from Spain revealed several conserved genomic features including an *ori* similar to that observed in begomoviruses, an adenine-rich region, and a γSCR. The γSCR is present in Ipomoea satellites and other gammasatellites associated with monopartite begomoviruses reported from Australia and Philippines. Although the γSCR is not present in all gammasatellite molecules, there is a core region (23 nt long) within the γSCR that is shared (87%–100% identity) among all gammasatellites reported to date, including sequences from geographically distant locations. This core region within the γSCR is similar to a region found within the βSCR, where gammasatellites and most betasatellites share ~87% identity. This supports the view that there is an evolutionary relationship between gammasatellites and betasatellites, as has been noted for gammasatellites identified in Cuba [57]. Future studies need to examine the prevalence of gammasatellites, their role in disease, if any, and their evolutionary history.

## 5. Conclusions

Since interactions among different begomoviruses and satellite DNAs may lead to the emergence of damaging diseases, it is critical to monitor begomovirus-satellite complexes present in crop fields and native vegetation. VEM using whiteflies is an unbiased sampling approach for detecting begomoviruses in different regions, providing an important glimpse into the reservoir of viral genetic diversity that is often overlooked due to the lack of visible symptoms. In this study, the VEM approach extended the known diversity and geographical range of satellite DNAs. This is the first report of alphasatellites from Central America and Ipomoea satellites from the Caribbean. Furthermore, the genetic information gathered led to the description of a phylogenetic clade (DNA-3-type alphasatellites) dominated by NW sequences and a third class of small, non-coding satellite molecules, named gammasatellites. The novelty of satellite DNAs described here suggests that these molecules hold unexplored genetic diversity. This is in contrast to recently described begomoviruses, which seem to represent variations of known genomic themes [37]. Therefore, satellite DNAs require further study as they may be a key factor driving the diversification of begomovirus-satellite disease complexes.

## Supplementary Files

Supplementary File 1

## References

[1] Leke W., Mignouna D., Brown J., Kvarnheden A. (2015). Begomovirus disease complex: Emerging threat to vegetable production systems of West and Central Africa. Agric. Food Secur..

[2] Zhou X. (2013). Advances in understanding begomovirus satellites. Annu. Rev. Phytopathol..

[3] Mansoor S., Briddon R.W., Zafar Y., Stanley J. (2003). Geminivirus disease complexes: An emerging threat. Trends Plant Sci..

[4] Mansoor S., Zafar Y., Briddon R.W. (2006). Geminivirus disease complexes: The threat is spreading. Trends Plant Sci..

[5] Ha C., Coombs S., Revill P., Harding R., Vu M., Dale J. (2008). Molecular characterization of begomoviruses and DNA satellites from Vietnam: Additional evidence that the New World geminiviruses were present in the Old World prior to continental separation. J. Gen. Virol..

[6] Nawaz-ul-Rehman M.S., Fauquet C.M. (2009). Evolution of geminiviruses and their satellites. FEBS Lett..

[7] Briddon R.W., Bull S.E., Amin I., Mansoor S., Bedford I.D., Rishi N., Siwatch S.S., Zafar Y., Abdel-Salam A.M., Markham P.G. (2004). Diversity of DNA 1: A satellite-like molecule associated with monopartite begomovirus-DNA β complexes. Virology.

[8] Xie Y., Wu P., Liu P., Gong H., Zhou X. (2010). Characterization of alphasatellites associated with monopartite begomovirus/betasatellite complexes in Yunnan, China. Virol. J..

[9] Romay G., Chirinos D., Geraud-Pouey F., Desbiez C. (2010). Association of an atypical alphasatellite with a bipartite New World begomovirus. Arch. Virol..

[10] Paprotka T., Metzler V., Jeske H. (2010). The first DNA 1-like α satellites in association with New World begomoviruses in natural infections. Virology.

[11] Briddon R.W., Stanley J. (2006). Subviral agents associated with plant single-stranded DNA viruses. Virology.

[12] Briddon R.W., Bull S.E., Amin I., Idris A.M., Mansoor S., Bedford I.D., Dhawan P., Rishi N., Siwatch S.S., Abdel-Salam A.M. (2003). Diversity of DNA β, a satellite molecule associated with some monopartite begomoviruses. Virology.

[13] Rosario K., Duffy S., Breitbart M. (2012). A field guide to eukaryotic circular single-stranded DNA viruses: Insights gained from metagenomics. Arch. Virol..

[14] Saunders K., Norman A., Gucciardo S., Stanley J. (2004). The DNA β satellite component associated with ageratum yellow vein disease encodes an essential pathogenicity protein (βC1). Virology.

[15] Saeed M., Behjatnia S.A.A., Mansoor S., Zafar Y., Hasnain S., Rezaian M.A. (2005). A single complementary-sense transcript of a geminiviral DNA β satellite is determinant of pathogenicity. Mol. Plant Microbe Interact..

[16] Cui X., Li G., Wang D., Hu D., Zhou X. (2005). A begomovirus DNA β-encoded protein binds DNA, functions as a suppressor of RNA silencing, and targets the cell nucleus. J. Virol..

[17] Eini O., Dogra S.C., Dry I.B., Randles J.W. (2012). Silencing suppressor activity of a begomovirus DNA β encoded protein and its effect on heterologous helper virus replication. Virus Res..

[18] Gopal P., Pravin Kumar P., Sinilal B., Jose J., Kasin Yadunandam A., Usha R. (2007). Differential roles of C4 and βC1 in mediating suppression of post-transcriptional gene silencing: Evidence for transactivation by the C2 of Bhendi yellow vein mosaic virus, a monopartite begomovirus. Virus Res..

[19] Sharma P., Ikegami M., Kon T. (2010). Identification of the virulence factors and suppressors of posttranscriptional gene silencing encoded by *Ageratum yellow vein virus*, a monopartite begomovirus. Virus Res..

[20] Patil B.L., Fauquet C.M. (2010). Differential interaction between cassava mosaic geminiviruses and geminivirus satellites. J. Gen. Virol..

[21] Idris A.M., Shahid M.S., Briddon R.W., Khan A.J., Zhu J.-K., Brown J.K. (2011). An unusual alphasatellite associated with monopartite begomoviruses attenuates symptoms and reduces betasatellite accumulation. J. Gen. Virol..

[22] Nawaz-Ul-Rehman M.S., Nahid N., Mansoor S., Briddon R.W., Fauquet C.M. (2010). Post-transcriptional gene silencing suppressor activity of two non-pathogenic alphasatellites associated with a begomovirus. Virology.

[23] Nawaz-ul-Rehman M.S., Mansoor S., Briddon R.W., Fauquet C.M. (2009). Maintenance of an Old World betasatellite by a New World helper begomovirus and possible rapid adaptation of the betasatellite. J. Virol..

[24] Saeed M., Zafar Y., Randles J.W., Rezaian M.A. (2007). A monopartite begomovirus-associated DNA β satellite substitutes for the DNA β of a bipartite begomovirus to permit systemic infection. J. Gen. Virol..

[25] Yang X., Xie Y., Raja P., Li S., Wolf J.N., Shen Q., Bisaro D.M., Zhou X. (2011). Suppression of methylation-mediated transcriptional gene silencing by βC1-SAHH protein interaction during geminivirus-betasatellite infection. PLoS Pathog..

[26] Zhou X., Xie Y., Tao X., Zhang Z., Li Z., Fauquet C.M. (2003). Characterization of DNA β associated with begomoviruses in China and evidence for co-evolution with their cognate viral DNA-A. J. Gen. Virol..

[27] Saunders K., Bedford I.D., Stanley J. (2002). Adaptation from whitefly to leafhopper transmission of an autonomously replicating nanovirus-like DNA component associated with ageratum yellow vein disease. J. Gen. Virol..

[28] Saunders K., Stanley J. (1999). A nanovirus-like DNA component associated with yellow vein disease of *Ageratum conyzoides*: Evidence for interfamilial recombination between plant DNA viruses. Virology.

[29] Singh A.K., Chattopadhyay B., Chakraborty S. (2012). Biology and interactions of two distinct monopartite begomoviruses and betasatellites associated with radish leaf curl disease in India. Virol. J..

[30] Kumar J., Kumar J., Singh S.P., Tuli R. (2014). Association of satellites with a mastrevirus in natural infection: Complexity of *Wheat dwarf India virus* disease. J. Virol..

[31] Huang C., Xie Y., Zhao L., Ren H., Li Z. (2013). A naturally occurring defective DNA satellite associated with a monopartite begomovirus: Evidence for recombination between alphasatellite and betasatellite. Viruses.

[32] Seal S.E., vandenBosch F., Jeger M.J. (2006). Factors influencing begomovirus evolution and their increasing global significance: Implications for sustainable control. Crit. Rev. Plant Sci..

[33] Harrison B.D., Robinson D.J. (1999). Natural genomic and antigenic variation in whitefly-transmitted geminiviruses (begomoviruses). Annu. Rev. Phytopathol..

[34] Rosario K., Capobianco H., Ng T.F., Breitbart M., Polston J.E. (2014). RNA viral metagenome of whiteflies leads to the discovery and characterization of a whitefly-transmitted carlavirus in North America. PLoS ONE.

[35] Ng T.F.F., Duffy S., Polston J.E., Bixby E., Vallad G.E., Breitbart M. (2011). Exploring the diversity of plant DNA viruses and their satellites using vector-enabled metagenomics on whiteflies. PLoS ONE.

[36] Ng T.F.F., Willner D.L., Lim Y.W., Schmieder R., Chau B., Nilsson C., Anthony S., Ruan Y.J., Rohwer F., Breitbart M. (2011). Broad surveys of DNA viral diversity obtained through viral metagenomics of mosquitoes. PLoS ONE.

[37] Rosario K., Seah Y., Marr C., Varsani A., Kraberger S., Stainton D., Moriones E., Polston J., Duffy S., Breitbart M. (2015). Vector-enabled metagenomic (VEM) surveys using whiteflies (Aleyrodidae) reveal novel begomovirus species in the New and Old Worlds. Viruses.

[38] Shatters R.G., Powell C.A., Boykin L., Liansheng H., McKenzie C.L. (2009). Improved DNA barcoding method for *Bemisia tabaci* and related Aleyrodidae: Development of universal and *Bemisia tabaci* biotype-specific mitochonddrial cytochrome c oxidase I polymerase chain reaction primers. J. Econ. Entomol..

[39] Inoue-Nagata A.K., Albuquerque L.C., Rocha W.B., Nagata T. (2004). A simple method for cloning the complete begomovirus genome using the bacteriophage φ29 DNA polymerase. J. Virol. Methods.

[40] Niu B., Fu L., Sun S., Li W. (2010). Artificial and natural duplicates in pyrosequencing reads of metagenomic data. BMC Bioinform..

[41] Altschul S.F., Madden T.L., Schaffer A.A., Zhang J.H., Zhang Z., Miller W., Lipman D.J. (1997). Gapped BLAST and PSI-BLAST: A new generation of protein database search programs. Nucleic Acids Res..

[42] Huson D.H., Mitra S., Ruscheweyh H.J., Weber N., Schuster S.C. (2011). Integrative analysis of environmental sequences using MEGAN4. Genome Res..

[43] Roux S., Tournayre J., Mahul A., Debroas D., Enault F. (2014). Metavir 2: New tools for viral metagenome comparison and assembled virome analysis. BMC Bioinform..

[44] Mubin M., Briddon R.W., Mansoor S. (2009). Complete nucleotide sequence of chili leaf curl virus and its associated satellites naturally infecting potato in Pakistan. Arch. Virol..

[45] Briddon R.W., Brown J.K., Moriones E., Stanley J., Zerbini M., Zhou X., Fauquet C.M. (2008). Recommendations for the classification and nomenclature of the DNA-β satellites of begomoviruses. Arch. Virol..

[46] Edgar R.C. (2004). MUSCLE: Multiple sequence alignment with high accuracy and high throughput. Nucleic Acids Res..

[47] Muhire B.M., Varsani A., Martin D.P. (2014). SDT: A virus classification tool based on pairwise sequence alignment and identity calculation. PLoS ONE.

[48] Tamura K., Peterson D., Peterson N., Stecher G., Nei M., Kumar S. (2011). MEGA5: Molecular evolutionary genetics analysis using maximum likelihood, evolutionary distance, and maximum parsimony methods. Mol. Biol. Evol..

[49] Abascal F., Zardoya R., Posada D. (2005). ProtTest: Selection of best-fit models of protein evolution. Bioinformatics.

[50] Guindon S., Dufayard J.-F., Lefort V., Anisimova M., Hordijk W., Gascuel O. (2010). New algorithms and methods to estimate maximum-likelihood phylogenies: Assessing the performance of PhyML 3.0. Syst. Biol..

[51] Anisimova M., Gascuel O. (2006). Approximate likelihood-ratio test for branches: A fast, accurate, and powerful alternative. Syst. Biol..

[52] Hodcroft E. TreeCollapser CL4. http://emmahodcroft.com/TreeCollapseCL.html.

[53] Tiendrebeogo F., Lefeuvre P., Hoareau M., Villemot J., Konate G., Traore A., Barro N., Traore V., Reynaud B., Traore O. (2010). Molecular diversity of *Cotton leaf curl Gezira virus* isolates and their satellite DNAs associated with okra leaf curl disease in Burkina Faso. Virol. J..

[54] Jeske H., Kober S., Schäfer B., Strohmeier S. (2014). Circomics of Cuban geminiviruses reveals the first alpha-satellite DNA in the Caribbean. Virus Genes.

[55] Rosario K., Padilla-Rodriguez M., Kraberger S., Stainton D., Martin D.P., Breitbart M., Varsani A. (2013). Discovery of a novel mastrevirus and alphasatellite-like circular DNA in dragonflies (Epiprocta) from Puerto Rico. Virus Res..

[56] Zaffalon V., Mukherjee S., Reddy V., Thompson J., Tepfer M. (2012). A survey of geminiviruses and associated satellite DNAs in the cotton-growing areas of northwestern India. Arch. Virol..

[57] Fiallo-Olivé E., Martínez-Zubiaur Y., Moriones E., Navas-Castillo J. (2012). A novel class of DNA satellites associated with New World begomoviruses. Virology.

[58] Dry I.B., Krake L.R., Rigden J.E., Rezaian M.A. (1997). A novel subviral agent associated with a geminivirus: The first report of a DNA satellite. Proc. Natl. Acad. Sci. USA.

[59] Mansoor S., Khan S.H., Bashir A., Saeed M., Zafar Y., Malik K.A., Briddon R., Stanley J., Markham P.G. (1999). Identification of a novel circular single-stranded DNA associated with cotton leaf curl disease in Pakistan. Virology.

